# The pneumococcal social network

**DOI:** 10.1371/journal.ppat.1008931

**Published:** 2020-10-29

**Authors:** Surya D. Aggarwal, Hasan Yesilkaya, Suzanne Dawid, N. Luisa Hiller

**Affiliations:** 1 Department of Biological Sciences, Carnegie Mellon University, Pittsburgh, Pennsylvania, United States of America; 2 Department of Respiratory Sciences, University of Leicester, Leicester, United Kingdom; 3 Department of Microbiology and Immunology, University of Michigan, Ann Arbor, Michigan, United States of America; 4 Department of Pediatrics, University of Michigan, Ann Arbor, Michigan, United States of America; University of Queensland, AUSTRALIA

## Abstract

Gram-positive bacteria employ an array of secreted peptides to control population-level behaviors in response to environmental cues. We review mechanistic and functional features of secreted peptides produced by the human pathogen *Streptococcus pneumoniae*. We discuss sequence features, mechanisms of transport, and receptors for 3 major categories of small peptides: the double-glycine peptides, the Rap, Rgg, NprR, PlcR, and PrgX (RRNPP)-binding peptides, and the lanthionine-containing peptides. We highlight the impact of factors that contribute to carriage and pathogenesis, specifically genetic diversity, microbial competition, biofilm development, and environmental adaptation. A recent expansion in pneumococcal peptide studies reveals a complex network of interacting signaling systems where multiple peptides are integrated into the same signaling pathway, allowing multiple points of entry into the pathway and extending information content in new directions. In addition, since peptides are present in the extracellular milieu, there are opportunities for crosstalk, quorum sensing (QS), as well as intra- and interstrain and species interactions. Knowledge on the manner that population-level behaviors contribute to disease provides an avenue for the design and development of anti-infective strategies.

## Introduction

Social behaviors are widespread across organisms. Ant colony formation, coordinated movement in locusts, and shoaling of fish are sophisticated examples of social behaviors. These behaviors benefit the population by providing protection against predation, increases in the food supply, or strategic advantages over competitors. Bacteria are no exception to such social behaviors. Bacteria perform quorum sensing (QS): cell density-linked signaling that results in the induction of a population-level response [1]. An early demonstration of QS was in the marine bacterium, *Vibrio fischeri*, where high cell density induces bioluminescence as part of a symbiotic relationship between bacteria and squid [2]. Since then, bacterial group behaviors have been implicated in cellular processes such as gene transfer, motility, antibiotic production, and biofilm formation [3–5].

Cell–cell communication is coordinated by signaling molecules that are secreted by the donor cell into the extracellular milieu and sensed by producing and neighboring cells [6]. Sensing results in changes in gene expression, ultimately triggering synchronized population behaviors. Central to this cell–cell communication is signaling by autoinducer-2 (AI-2) and peptides. This review highlights mechanistic and functional features of secreted cell–cell communication peptides produced by the human pathogen *Streptococcus pneumoniae*.

*S*. *pneumoniae* (or pneumococcus) is a major cause of otitis media, bacterial meningitis, septicaemia, and community-acquired pneumonia. WHO classifies pneumococcus as an antibiotic-resistant “priority pathogen.” It is 1 of the top causes of lower respiratory infections and is responsible for almost 1 million annual deaths in children worldwide [7–9]. The pneumococcus occupies different niches in the human host leading to commensal and pathogenic existences; these occupations can occur sequentially and/or simultaneously in the form of biofilm or planktonic modes of growth. Invasive pneumococcal disease is a multistep process; it is initiated through pneumococcal infiltration into the sugar-rich mucus layer, followed by adherence to the epithelial cell layer of the human nasopharynx. Often, the microbe colonizes the nasopharynx for an extended period of time without causing disease. Alternatively, for reasons unknown, it can disseminate into the middle ear, lungs, brain, or blood. Entry into the blood, either directly from the nasopharynx or most often through lungs, provides access to the central nervous system, the heart, and the spleen. In all these tissues, the pneumococcus is exposed to assaults by the immune system and diverse environmental conditions including varying concentration of oxygen, metals, sugars, and a wide temperature range [10–17]. In such fluctuating host environments, community-level synchronization of transcription enables microbes to acquire a phenotype consistent with the requirements of the specific host niche. In this manner, peptide-mediated communication is critical to colonization and pathogenesis.

### Overall classification of pneumococcal cell–cell communication systems

**Fig 1:**
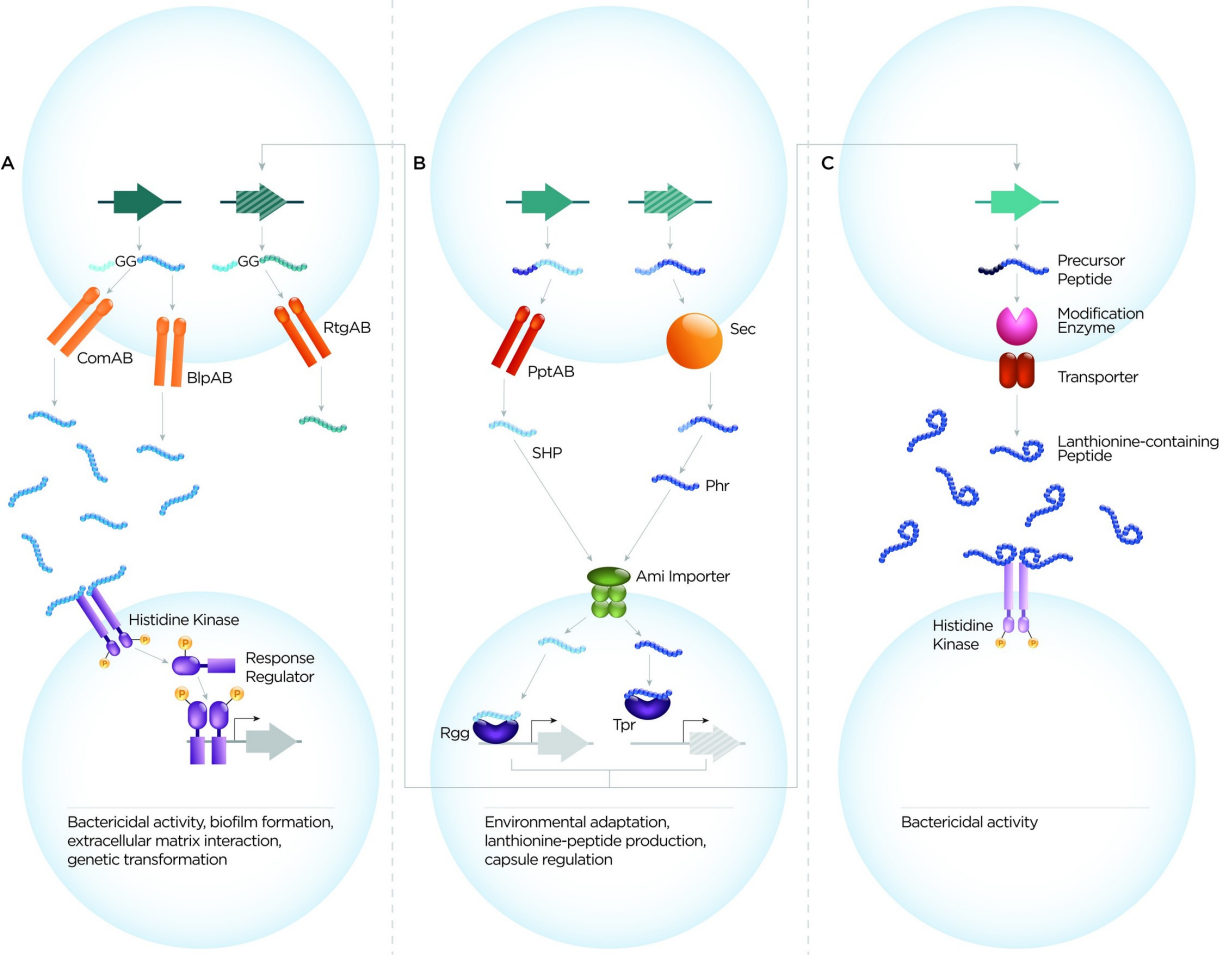
Schematic diagram of 3 main classes of pneumococcal secreted peptides. Schematic diagram showing peptide features, processing and export, receptors, and phenotypic consequences of different families of peptides, namely (A) double-glycine peptides, (B) peptides signaling via the RRNPP superfamily of regulators, and (C) lanthionine-containing peptides. **(A)** The conserved N-terminal leader of double-glycine peptides guides them for processing and export via C39-peptidase domain containing ABC transporters, ComAB, BlpAB, or RtgAB. A combination of features in both the leader sequence and cargo peptide determine transporter-substrate specificity and efficiency of transport. Some peptides can be exported by multiple transporters. The secreted peptide can activate a response in the recipient cell either directly (e.g., fratricide by CibAB) or indirectly by inducing signaling upon binding a receptor (e.g., CSP and BIP). Double-glycine peptides lead to downstream phenotypes that include biofilm formation, extracellular matrix interaction, genetic diversity, and bactericidal activity. **(B)** Peptides that signal via the RRNPP superfamily of regulators include SHP and Phr peptides. Based on data for 1 pneumococcal SHP (RtgS) and from other species, we propose that SHPs are secreted outside the cell by the ABC transporter, PptAB. It is proposed that SHPs undergo processing and maturation either concomitant with (via the Eep membrane protease) or after their secretion (via an uncharacterized protease). The mechanisms of Phr peptide processing in pneumococcus remain unknown. Following maturation, both SHP and Phr peptides are imported into the recipient cell via an oligopeptide permease system, AmiACDEF. Once internalized, SHPs interact with their cognate Rgg regulators resulting in DNA binding and transcriptional activation. Phr peptides interact with their cognate Tpr regulators, releasing Tpr-mediated inhibition of gene expression. Signaling via SHP and Phr peptides facilitates environmental adaptation for the bacteria and can induce production of double-glycine and lanthionine-containing peptides. **(C)** The lanthionine-containing peptides undergo posttranslational modifications and cyclization by the action of intracellular lanthionine modification enzymes. Owing to their conserved leader sequence, these propeptides are directed for secretion by dedicated ABC transporters. While the targets and function (as signal or bacteriocins) of most lanthionine-containing peptides are unknown, pneumolancidin functions by activating a TCS system on the target cells, ultimately exerting bactericidal activity. ABC, ATP-binding cassette; BIP, bacteriocin-inducing peptide; CSP, competence-stimulating peptide; GG, double-glycine; Phr, phosphatase regulator; Rgg, Regulator gene of glycosyltransferase; RRNPP, Rap, Rgg, NprR, PlcR, and PrgX; SHP, short hydrophobic peptide; TCS, two component systems.

The pneumococcal cell–cell communication systems can be classified into 3 main categories based on peptide sequences, transporters, and receptors (**Fig 1**). These are (1) double-glycine peptides, (2) peptides associated with the RRNPP superfamily of QS proteins, and (3) lanthionine-containing peptides. In terms of their roles, the peptide-mediated cell–cell communication systems fulfill at least 3 main functions: of ensuring genetic diversity, microbial competition, and environmental adaptation.

#### Double-glycine peptides

The best characterized pneumococcal double-glycine peptide is the competence-stimulating peptide (CSP) [18]. Other examples include the bacteriocin-inducing peptide (BIP) [19], the competence-induced bacteriocins (CibA and CibB) [20], peptides of the bacteriocin immunity region (BIR) [21], the virulence peptide 1 (VP1) [22], the biofilm-regulating peptide induced by competence (BriC) [23,24], peptides of the *rtg* locus [25], and LanA [22,26] (**Table 1, S1 Table**). Furthermore, comparative genomic approaches have revealed additional double-glycine peptides that remain to be characterized [22,27].

**Table 1:** Ribosomally synthesized peptides experimentally studied in *Streptococcus pneumoniae*. Peptides are divided into different families: (1) Double-glycine peptides (green), (2) RRNPP peptides—small hydrophobic peptides (dark blue) & Phr peptides (light blue), and (3) lanthionine-containing peptides (yellow). Gene ID for TIGR4 (*sp*_) and D39 (*spd*_).

S. No.	Peptide	Gene Number	Exporter	Receptor (if Signaling)	Strain Distribution^#^	Function	References
1	CSP	*sp_2237 (spd_2065)*	ComAB and BlpAB	ComD	Core or Almost Core	Competence	[18,30,35]
2	CibA and CibB	*sp_0125 (spd_0133)* and *sp_0124 (spd_0132)*	ComAB* and BlpAB*		Core or Almost Core	Fratricide	[20]
3	BriC	*sp_0429* (*spd_0391*)	ComAB	Unknown	Core or Almost Core	Biofilm Development and Colonization	[23,24]
4	BIP	*sp_0528* (*spd_0470)*	BlpAB and ComAB	BlpH	Core or Almost Core	Bacteriocin Production	[19,31,32,33]
5	BlpM and BlpN	*sp_0539* and *sp_0540*	BlpAB* and ComAB*		Accessory	Bacteriocin	[21,31]
6	BlpI and BlpJ	*sp_0531* and *sp_0532*	BlpAB and ComAB		Accessory	Bacteriocin	[21,36]
7	BlpK	*sp_0533* and *sp_0041* (*spd_0046*)	BlpAB* and ComAB*		Accessory	Bacteriocin	[21,36]
8	VP1	*sp_0142* (*spd_0145*)	Unknown	Unknown	Core or Almost Core	Adherence to Epithelial Cells and Colonization	[22,79]
9	RtgG	*CGSSp9BS68_07272*	RtgAB	Unknown	Rare	Unknown	[25]
10	RtgC	*sp_0115* (*spd_0116*)	RtgAB	Unknown	Accessory	Unknown	[25]
11	RtgT	*CGSSp9BS68_02658* (or *spd_0121*)	RtgAB*	Unknown	Accessory	Unknown	[25]
12	RtgW	*CGSSp9BS68_07277* (or *spd_0123*^*##*^)	RtgAB*	Unknown	Accessory	Unknown	[25]
13	SHP144	Adjacent to *sp_0141* (*spd_0144*)	PptAB*	Rgg144	Core or Almost Core	Environmental Adaptation	[22,43]
14	SHP939	Adjacent to *spd_0939*	PptAB*	Rgg939	Accessory	Environmental Adaptation	[42,43]
15	RtgS (Type A and B)	Adjacent to *sp_0114* (*spd_0112* or *CGSSp9BS68_07247)*	PptAB	RtgR	Accessory	Colonization	[25]
16	SHP1518	Adjacent to *spd_1518*	PptAB*	Rgg1518*	Accessory	Unknown	[25,43]
17	PhrA	*sp_1947* (*spd_1746*)	Sec secretion pathway	TprA	Accessory	Virulence	[26]
18	PhrA2	*spn23F_12740*	Sec secretion pathway	TprA2	Rare (Unique to PMEN1)	Commensalism	[44]
19	LanA1^ and LanA2^	*sp_1948* (*spd_1747*) and *sp_1949 (spd_1748)*	LanT*		Accessory	Unknown	[22,26]
20	LcpA	*spn23F_12701*	LcpT*		Accessory	Unknown	[44]
21	PldA1–PldA4	Downstream of the *spn23F_12760* Homolog in P174	PldT		Rare	Bacteriocin	[66]

*Putative, lacks direct experimental evidence.

^#^Categorized based on presence in strains tested in published data sets above in addition to Javan et al. [27]: Rare: Less than 10%, Accessory: 10%–90%, Core or Almost Core: More than 90%.

^##^Contains an alternative start codon 33 bp upstream in D39.

^^^LanA peptides also contain N-terminal leader sequences characteristic of double-glycine peptides.

BIP, bacteriocin-inducing peptide; BriC, biofilm-regulating peptide induced by competence; CSP, competence-stimulating peptide; Phr, phosphatase regulator; PMEN1, pneumococcal molecular epidemiology network clone 1; RRNPP, Rap, Rgg, NprR, PlcR, and PrgX; VP1, virulence peptide 1.

Double-glycine peptides are characterized by a conserved N-terminal leader sequence that terminates in Gly–Gly residues (or more rarely in Gly–Ala or Gly–Ser) [22,28] (**Fig 1A**). The leader guides these peptides to peptidase-containing ATP-binding cassette (ABC) transporters (with a C39-peptidase domain), which cleave the leader sequences and export peptides out of the cell [29]. The genomic locus of CSP and BIP encodes their cognate exporters ComAB and BlpAB, respectively [29–31]. A frameshift mutation renders BlpAB nonfunctional in approximately 60% of the strains. Yet, during competence activation, these strains employ ComAB for BIP secretion, illustrating redundancy in exporters [21,32–34]. Further, ComAB also contributes to the export of BriC [23]. However, not all transporters are promiscuous. Peptides of the *rtg* locus are not secreted by ComAB or BlpAB and instead are exported by another transporter, RtgAB [25]. Finally, BlpI, a peptide from BIR, can be secreted by all 3, albeit at different efficiencies. It appears that a combination of features in the leader sequence and the cargo peptide determine the nature of the transporter and efficiency of transport. The entire rulebook of transporter substrate specificity is a subject of intense research, and once resolved will facilitate in silico prediction of peptide transporter sets.

Once secreted from the donor cell, some double-glycine peptides induce a response in the recipient by signaling through two component systems (TCS). The peptide binds the histidine kinase of the TCS, triggering its autophosphorylation and the transphosphorylation of its cognate response regulator, subsequently altering the transcriptional state of the cell. This is observed for signaling of CSP via ComDE and of BIP via BlpHR, where peptide–receptor pairs are located in the same locus [19,35]. Other double-glycine peptides are not adjacent to TCS systems, and as such, their receptors have not been identified; they may induce signaling via TCSs or other families of receptors. For the double-glycine peptides with bacteriocidal activities, such as CibAB and the peptides encoded within the BIR, it remains unclear whether the bacteriocidal activity even requires binding partners [20,31,36]. Finally, LanA possesses features of both double-glycine and lanthionine-containing peptides (see below), and it is unclear the extent to which its transport and receptors (if any) share features with each of these families. Studies on double-glycine peptides have focused on their role across bacteria, emerging evidence suggests that the host may also be listening. A G-protein couple receptor on mast cells binds positively charged peptides, including CSP, triggering an immune response and enhancing bacterial clearance [37].

#### Peptides of RRNPP superfamily of QS proteins

Peptides in this group signal by direct interaction with their cognate cytoplasmic transcription factors, which are members of the RRNPP superfamily [38–40] (**Fig 1B**). These peptides can be classified based on a variety of sequence features, as previously reviewed [41]. In pneumococcus, the short hydrophobic peptide (SHP) SHP144, SHP939, SHP1518, and RtgS, and the phosphatase regulator (Phr) peptides PhrA and PhrA2 have been characterized [22,25,26,42–46].

Across streptococci, Regulator gene of glycosyltransferase (Rgg) proteins are activated upon binding with their cognate SHP, which are usually encoded adjacent to *rgg* genes [41,47,48]. SHPs are only active after export, after processing from precursor polypeptides that are typically shorter than 35 residues [39,49]. The processing protease(s) have not been studied in pneumococcus, yet in other streptococcal species, a membrane-bound metalloprotease (Eep) contributes to the processing [40,50]. Multiple streptococcal species export SHPs through an ABC transporter, PptAB, whose homolog in *Enterococcus faecalis* exports sex pheromones [51–53]. In the absence of a known target sequence, the mechanisms directing SHPs to their transporter remain unclear. The mature peptides are reimported into the cell via an oligopeptide permease system, where they interact with the cognate Rgg regulator altering the cell’s transcriptional state [40,50,52]. In pneumococcus, RtgS is the only SHP whose precursor peptide has been shown to be exported by PptAB and internalized by the Ami oligopeptide importer, AmiACDEF [25]. Given the broad function of PptAB across species, it is likely that the other pneumococcal SHPs utilize the same mechanism for export and import.

Phr peptides signal through members of the RRNPP superfamily [54,55]. In *Bacillus subtilis*, export of Phr precursor peptides is mediated by a conserved N-terminal signal sequence that directs export via the Sec-dependent pathway [55,56]. Additional maturation takes place extracellularly to generate 5 to 7 residues peptides, which are internalized by oligopeptide permease systems [55–61]. The pneumococcal pangenome encodes at least 4 Phr peptides (PhrA, PhrB, PhrC, and PhrA2), of which PhrA and PhrA2 have been characterized experimentally [26,44–46]. While the Phr peptides in *Bacilli* signal by interacting with Rap proteins, the pneumococcal Phr peptides interact with the Tpr (transcription factor regulated by Phr peptide) set of PlcR homologs [26,55]. The genomic organization of pneumococcal Phr-signaling cassettes is in contrast with those encoded in *B*. *subtilis*, since the pneumococcal *tprA (*and *tprA2)* and *phrA (*and *phrA2)* are oriented in opposite directions and not the same direction [26]. However, like other species, the pneumococcal PhrA is internalized in the cell through the oligopeptide permease system, AmiACDEF [26]. Once internalized, PhrA and PhrA2 interact with their cognate Tpr regulators and relieve the Tpr-mediated inhibition of gene expression.

#### Lanthionine-containing peptides

This is a family of small (19–38 amino acids) peptides produced by gram-positive bacteria that possess diverse structures and functions. These are cyclic peptides, characterized by posttranslational modifications that result in the introduction of thioether amino acids lanthionine and methyllanthioine (**Fig 1C**) [62]. Their characteristic structure is formed by LanM modification enzymes, when serine or threonine residues in the propeptide are dehydrated and linked to cysteine thiols. The peptide is exported by dedicated LanT transporters [62–64]. Many lanthionine-containing peptides form 1 of the 2 major classes of bacteriocins [65]. These peptides are known as lantibiotics, or lanthionine-containing antibiotics [62]. Genes for processing of lanthionine-containing peptides, modification enzymes, immunity proteins, and transporters are generally organized in clusters. Numerous such clusters are present in pneumococcus; of these, the lanthipeptides associated with Tpr/Phr and the pneumolancidin cluster have been studied [26,44,66].

### Functional attributes of pneumococcal cell–cell communication systems

The pneumococcal peptide mediated cell–cell communication systems provide several, not mutually exclusive, functionalities. A cell–cell communication system can be conceptualized as a circuit that controls population-level structures and behaviors. Many of these cell–cell communication circuits respond to diverse environmental stimuli such as population density, nutritional status, pH, oxygen availability, and antibiotic stress [67–74]. Signaling from cell–cell communication systems phenotypically converge in changes at the population level. These may impact development of biofilms or be associated with modifications in cell surface components such as membrane composition and capsule expression [22,23,43]. Cell–cell communication system behaviors may also be accompanied by modification in ability for DNA uptake, fratricide, or bactericidal activity [20,23,31,75,76]. These physiological changes may alter the propensity of cells to acquire antibiotic resistance genes and influence the emergence of vaccine-escape strains. Further, intercellular communication systems may also regulate degradation of host matrix, biofilm development, and nutrient uptake ability [22,23,43,77–79]. Thus, together these properties influence pathogenic potential, antibiotic resistance, and response to vaccines.

The ability to regulate population responses provide competitive advantage to pneumococcal cells over other microbial species inhabiting nasopharynx. Cell–cell communication systems enable the microbe to alter its transcriptional profile to acquire a suitable phenotype to optimize the population-level fitness. It is very likely that the systems such as competence, which introduce diversity at the DNA level and transcriptional level, enable long-term maintenance of commensal lifestyle in the nasopharynx, where the pneumococcus is found in biofilms in highly variable densities during asymptomatic periods [80]. Further, in general, “transcriptional adaptation” not only contributes to survival in the dynamic nasopharynx but also promotes survival when the microbe migrates from 1 host niche to another during infection.

#### Generation of genetic and phenotypic diversity

Competence cascade is 1 of the key mechanisms for generating both genetic diversity by DNA acquisition and phenotypic diversity by changes in gene expression. Nonmutually exclusive hypotheses have been proposed to explain how DNA acquisition provides a fitness advantage: genomic diversification, nutrient source, and DNA for repair. The activation of competence pathway in response to DNA damage has also been described as a general stress response phenomenon [76].

**Fig 2:**
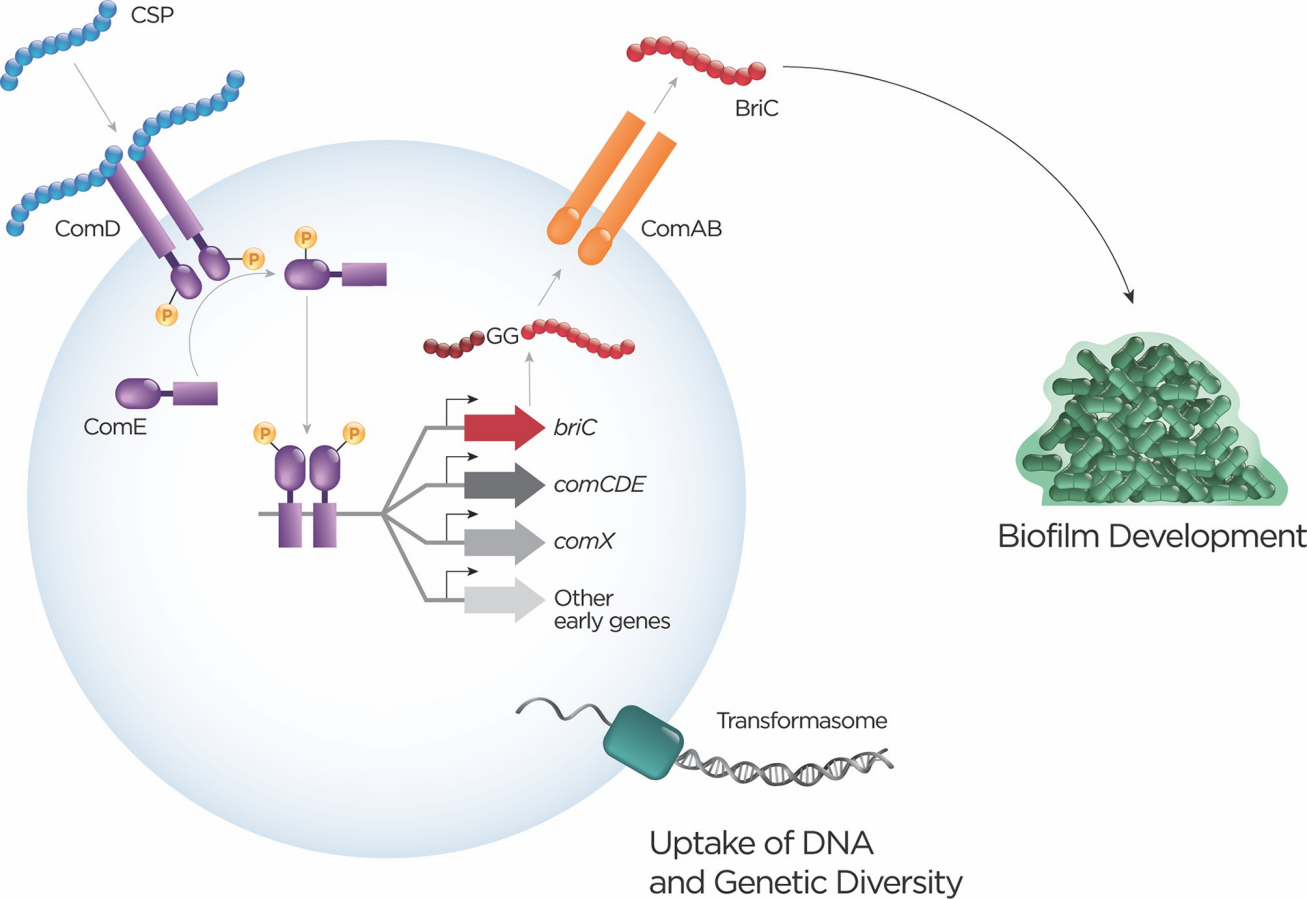
Hierarchical activation of multiple double-glycine peptides. The competence pathway is turned on by activation of ComDE by the double-glycine peptide, CSP. Competence induction by CSP results in transcriptional activation of a number of double-glycine peptides including BIP, CibAB, and BriC, each of which have phenotypic consequences. Activation of the competence pathway also allows for uptake of DNA and generation of genetic diversity. The schematic diagram illustrates ComE-dependent induction of *briC* along with other early competence genes. Upon being exported through ComAB, intercellular communication via BriC promotes biofilm development. This highlights the interconnectedness of mechanisms that impart genetic and phenotypic diversity. The design architecture that allows for hierarchical activation of different intercellular communication peptides may provide an opportunity for alternative activation of subsets of genes without expending energy to turn on the entire pathway. BIP, bacteriocin-inducing peptide; BriC, biofilm-regulating peptide induced by competence; competence-induced bacteriocin; CSP, competence-stimulating peptide; GG, double-glycine; TCS, two component systems.

The competence system is activated upon detection of the CSP: the canonical representative of the double-glycine peptide family. The pneumococcal pangenome possess 6 diverse alleles for *comC*, where the majority of strains encode 1 out of 2 allelic variants [81]. In a mixed population, competence may not only be spatially localized to a certain region within a biofilm; signals may be confined to individual pherotypes **(Box 1)**. CSP is autoinduced by cues including high cellular density, increase in pH, oxygen availability, and antibiotic stress [68,69,71–74]. In addition to diffusing through the environment, CSP signals to the neighboring cells through other mechanisms that include autocrine signaling and cell–cell contact [74,82,83]. Activation results in transcriptional changes in up to 10% of a strain’s genes [75,84]. The best characterized response is activation of the transformation machinery, allowing incorporation of foreign DNA by recombination, and, in doing so, contributing to generation of genetic diversity by exchange of alleles and changes in gene possession **(Fig 2)**. The exchange of genetic material within the pangenome tests novel genetic combinations where individual alleles or gene fragments have already overcome pruning by selection.

**Box 1. Pherotypes**

The term “pheromone” was coined in 1959 to refer to secreted substances that mediate intraspecies communication resulting in specific actions such as behavioral changes [120]. “Pherotypes” are pheromone signal variants generated due to allelic differences, and a pheromone may possess multiple distinct pherotypes. These pherotypes confer the cells with a mechanism for providing specificity in signal–receptor interactions. However, there is no difference in the molecular pathway that is regulated downstream of the receptors. Pherotypes are characterized by amino acid variations in certain key residues of the functional peptide pheromone that are important for interactions with the receptor. Mutations in other residues that are not important for this interaction will not lead to generation of a distinct pherotype. Allelic changes that result in the generation of pherotypes are usually accompanied by corresponding changes in the sequence of their cognate receptors to maintain the specificity of pherotype–receptor interactions and enable strain-specific signaling. The best characterized examples of pneumococcal pherotype–receptor pairs are that of CSP–ComD and BIP–BlpH [81,92,121]. Other signaling peptides such as VP1 and BriC also encode for multiple alleles and suggest the existence of pherotypes, yet their cognate receptors have not been discovered [22,23].

A pneumococcal cell encodes for multiple peptides that mediate intercellular communication. Strains encode for a distinct pherotype for each of the peptides they possess. As such, this has given rise to a diversity of pherotype combinations among the strains at the population level. For each of these peptides, strains that respond to 1 pherotype are grouped together into a “pherogroup” for the given peptide. While 2 strains may belong to the same pherogroup for peptide A, they may be placed in different pherogroups for peptide B. For instance, even though BriC is induced upon CSP-mediated competence activation, there is no association between CSP pherotypes and alleles encoding for BriC among pneumococcal strains [23]. Such an arrangement allows for an additional level of control during intraspecies kin identification. Alternatively, nonidentical distribution of strains into distinct pherogroups for different peptides may expand the repertoire of intraspecies interactions. This may be especially important during episodes of multistrain infection. Further, this also presents an opportunity for granularity in the regulation of a subset of genes within the cell. In the example above, BriC may serve as an additional point of entry into regulation of a subset of genes in the cell. This form of regulation may be efficient for a population, wherein the cells can optimally expend their energy in carrying out the desired phenotypic effect.

Given the existence of different pherotypes of CSP in pneumococcus, an intriguing question was whether these polymorphisms in CSP pherotypes alter the pneumococcal population structure. It was hypothesized that existence of pherotypes may facilitate recombination among strains belonging to the same CSP pherogroup, resulting in the maintenance of a genetically diverse subpopulation within the species [122,123]. However, mounting evidence has suggested that there is no increased genetic exchange between strains belonging to the same CSP pherogroup. Instead, CSP-mediated fratricide facilitates recombination between strains with varying CSP pherotypes [124,125].

Competence activates BriC, a widespread double-glycine peptide, up-regulated during the early phase of competence activation [23,24]. In addition to the competence-dependent regulation, a subset of clinically important pneumococcal strains contains a promoter insertion (RupB1) that provides for a competence-independent mechanism of *briC* induction. BriC stimulates biofilm development and colonization in a murine model. A pathway that connects competence and biofilm development increases opportunities for DNA uptake and generation of diversity **(Fig 2)**. There are 19 different alleles of *briC* in the pneumococcal population, yet most strains encode for 1 of 2 major alleles. Interestingly, there is no clear correlation between CSP pherotypes and *briC* alleles. Finally, a murine study analyzing evolution of pneumococcal population during colonization of a single strain captured nonsynonymous mutations in coding sequence of *briC* that were fixed in the population. These data suggest that *briC* is either linked to a genotype under selection or that *briC* itself is under selective pressure during long-term carriage [85].

#### Microbial competition

Intra- and interspecies competition is part of the pneumococcal lifestyle in the respiratory tract. Bactericidal activity is generated through small molecules from the double-glycine and lanthionine-containing peptide families [20,31,36,66]. The ability to restrict the growth of competitors is important for bacterial colonization. Moreover, victims may serve as a source of DNA, increasing the potential for evolution.

Competence activation leads to the production of many of these effector molecules, including CibAB and BIR locus. The double-glycine peptides CibAB are triggers for allolysis or lysis *in trans* and are responsible for lysis of noncompetent cells in a cell contact–dependent manner [20]. Similar to the *Lactococcus* bacteriocin IFPL105, this fratricide is thought to be carried out by an insertion of the bacteriocin in the membrane of sensitive cells leading to a depletion of their cellular energy [86,87]. Thereafter, lysis is caused by the action of cell wall hydrolases that include autolysin LytA, lysozyme LytC, and murein hydrolase CbpD [20,87]. The transmembrane peptide CibC protects cells against allolysis by CibAB [87]. The nutrients and DNA released from noncompetent cells by CibAB may benefit the attacking cells [87]. In a murine model of colonization, the allolysis induced by CibAB provides resident strains the ability to resist competition and colonization by invading strains [88].

The BIR encodes a wide variety of effector genes that confer either bactericidal activity or immunity from their inhibitory action [21]. The BIR locus is syntenic, yet the bacteriocins (putative and characterized) and immunity proteins vary extensively across strains. The products of *blpIJ*, *blpMN*, and *blpK* have confirmed bactericidal activity. These are double-glycine peptides, exported by BlpAB, and cotranscribed with cognate immunity proteins [31,36]. All these peptides display interstrain activity, and, in vivo, the BlpMN and BlpIJ bacteriocins provide strains with a competitive advantage over immunity-deficient strains during colonization [25,31]. Beyond pneumococcus, expression of the bacteriocin locus also inhibits some other gram-positive bacteria including *Streptococcus pyogenes*, *Streptococcus mitis*, *Streptococcus oralis*, and *Lactococcus lactis* but not others such as *Streptococcus mutans*, *E*. *faecalis*, or *Listeria monocytogenes* [36].

The expression of the BIR loci is induced by BIP, which is encoded by *blpC* and upstream of BIR [19,36,89–91]. Analogous to CSP, BIP binds a membrane-bound histidine kinase (BlpH), and there is specificity between the peptide and its receptor [92]. This specificity restricts crosstalk between competing pherotypes. Similar to the activation of the competence pathway, expression of BIP is induced by antibiotics and increases in pH [32]. Moreover, there is crosstalk between the competence and bacteriocin systems, wherein the production of BIP is induced upon CSP stimulation [33].

Another class of bacteriocins is the lanthionine-containing peptides, of which pneumolancidin (*pld*) is characterized [66]. The *pld* locus is rare across pneumococcal isolates and characterized by 4 tandem putative short peptide homologs (PldA1–PldA4). Three of these 4 peptides, PldA1–3, are required for the cell’s bactericidal activity, while the fourth peptide PldA4 is dispensable for the phenotype [66]. In addition to their bactericidal properties, pneumolancidins (PldA1–3) serve as autoinducing signaling peptides that signal through the histidine kinase encoded within the locus (*pldK*) resulting in activation of the *pld* locus [66]. Immunity is conferred by a neighboring ABC transporter, PldFE. The signaling and bactericidal role of these peptides are interconnected: when signaling by Pld peptides is low, PldA2 does not induce bacterial inhibition [66]. In addition, pneumolancidins provide pneumococcal strain with a competitive advantage during colonization in mice [66].

Finally, a comprehensive comparative genomic screen reveals many other peptides, with varied distributions across pneumococcal strains (from rare to core), as well as unique to pneumococcus or shared across streptococcal species [27]. Many are organized into operons with putative transporters, modification proteins, or immunity proteins. The diverse distributions within pneumococcus strains and related species is consistent with roles in intra- and interspecies microbial competition.

#### Impact of cell–cell communication systems on environmental adaptation

The pneumococcus has a network of cell–cell communication systems that modulate its adaptation to the host environment. Pneumococcus can only use sugars for the generation of its metabolic energy [93]. Further, sugars are used for capsule production and perhaps signaling [94]. In addition, transport of sugars by phosphotransferase systems (PTS) can trigger phosphorylation-dependent signal pathways [95,96]. Moreover, degradation of host sugars is not only a source of nutrients but also a major contributor to host adhesion, colonization, and virulence.

The expression of several cell–cell communication peptides is responsive to levels of host carbohydrates: SHP144 and SHP939 are induced in mannose and galactose and PhrA in galactose. Further, these peptides, as well as PhrA2 and VP1, are repressed in rich media [22,43,44,46]. The Rgg144/SHP144 system is core; it is activated when the autoinducing peptide SHP144 is imported into the cell and binds Rgg144. Rgg144 is negatively controlled by master nutritional regulator CodY and glutamine/glutamate metabolism [97,98]. It has an extensive regulon, which includes regulation of genes that have a function in replication, recombination, translation, and nucleotide transport and metabolism [43]. The most highly induced locus encodes the double-glycine signaling peptide VP1. It also controls multiple sugar transporters and represses capsule biosynthesis [43,79]. Further, the regulon is sugar specific, with a broad response in mannose and a restricted response in galactose. The mechanism underlying this sugar-specific regulation is unknown but may emerge from a regulatory web that includes multiple Rggs.

SHP939 is an autoinducing peptide that positively regulates Rgg939; this system is part of the accessory genome [42,43]. The diversity of genes regulated by Rgg939/SHP939 varies in response to environmental conditions, with an extensive regulon when grown on mannose and a limited regulon in galactose [42,43]. The regulon is predicted to function in cell division, iron transport, cell membrane biogenesis, and metabolism among others. Rgg939 also regulates capsule synthesis. However, both negative and positive regulation have been reported in strain D39, and the factors responsible for the differences remain to be determined [42,43]. Zhi and colleagues suggest that Rgg939 can directly inhibit expression from the capsule locus [43]. Related to this, Roger Junges and colleagues showed that Rgg939 expression positively regulates the expression of genes involved in production of compounds present in capsular polysaccharides of serotypes 12A and 12F, but outside the capsular locus [42]. Rgg939/Shp939 signaling negatively regulated biofilm development on A549 epithelial cells [42]. In mice, Rgg939/SHP939 was shown to be associated with a reduction in bacterial fitness in a pneumonia model [42], or alternatively, increased nasopharyngeal colonization and virulence in carriage and pneumonia models [43].

The Rgg systems do not act in isolation, but instead appear to form a connected network. In accordance, the presence of noncognate Rgg regulators is required for maximal induction of SHP144 and SHP939 [43]. Further, Rgg1518 controls its adjacent locus (SPD_1513–1517), and these genes are also regulated by Rgg144 and Rgg939 [43]. This crosstalk may extend beyond the boundaries of species. The sequence of SHP939 is identical to that of SHP3 found in *S*. *pyogenes* and differs by 1 residue from SHPs in *Streptococcus agalactiae* and *S*. *mitis* [42,48,99]. This similarity in SHP sequences may allow pneumococcal Rgg systems to be impacted by other resident bacteria.

The web of Rgg regulators and associated peptides extends beyond the RRNPP peptides and includes double-glycine peptides. Rgg144 is a positive inducer of VP1, and the Rgg-regulated transporter of double-glycine peptides (RtgR) is a positive inducer of a locus which encodes multiple double-glycine peptides. In these instances, the Rgg and peptides are adjacently located on the genome [22,25,43]. The gene *rgg144* is down-regulated by CodY, suggesting that once CodY-mediated inhibition is relieved, Rgg144 enhances transcriptional activation of *vp1*. VP1, present in the vast majority of strains, is 1 of the most highly expressed transcripts when pneumococci are exposed to host cells [22,24]. Functionally, VP1 promotes biofilm development and positively regulates a genomic locus responsible for processing, import, and catabolism of hyaluronic acid [79,100,101]. In animal models, VP1 is a virulence factor that contributes to carriage, dissemination, and mortality [22,79].

RtgR is encoded with a SHP (RtgS) and an ABC transporter. RtgS activates RtgR, and some strains encode 2, highly similar, copies of *rtgS*. The transporter secretes double-glycine peptides localized downstream **(Table 1, S1 Table)** [25]. There is high diversity in the distribution of *rtg*-associated double-glycine peptides present across the pneumococcal population: there is variability in the encoded peptides as well as the number of peptides present in a strain. The function of these peptides is unclear, but a functional *rtg* locus provides a competitive advantage during colonization of the mouse nasopharynx.

The PlcR family is a second family of peptide/regulator systems regulated in response to nutritional sensing and induced in vivo [44]. The core TprA/PhrA and the pneumococcal molecular epidemiology network clone 1 (PMEN1)-associated TprA2/PhrA2 systems induce Phr and lanthionine-containing peptides. The Phr peptides bind to their cognate Tpr regulator. The *phrA* promoter contains a catabolite-responsive element (CRE) region for binding by carbon-catabolite repressor CcpA [102]. In high glucose, binding of CcpA to the *phrA* promoter inhibits its transcriptional activation. Instead, *phrA* is expressed in the presence of galactose and mannose [26,45,46]. GlnR, a regulator of glutamine and glutamate metabolism, also binds the promoter region of *tprA* activating its expression in the presence of multiple different sugars [46]. Thus, activation of the TprA/PhrA signaling cascade is regulated by a complex network of different metabolic pathways.

After its import, the mature PhrA peptide binds to TprA regulator releasing it from the DNA. TprA is usually a negative regulator, such that PhrA promotes gene expression. This includes genes involved in sugar metabolism, neuraminidase, genes neighboring and regulated by Rgg1518, and a locus for the synthesis and processing of lanthionine-containing peptides [26,45]. In an in vivo setting, the high abundance of galactose and mannose in the host glycans and the nasopharynx [14,15] are expected to turn on the TprA/PhrA signaling system. PhrA promotes virulence in a chinchilla otitis media model and murine models of pneumonia and septicemia [45,103]. Evidence that anti-peptide therapies may be effective in combating pneumococcal disease comes from anti-PhrA peptides, where soluble linear molecularly imprinted polymers (LMIP) targeting PhrA dramatically decreased morbidity in mice [45]. It is curious that the TprA/PhrA signaling system impacts virulence in the blood, where glucose is in abundance. Perhaps, in this setting, the impact on TprA/PhrA system may be attributed to other pathways and not to galactose and mannose metabolism [46].

**Fig 3:**
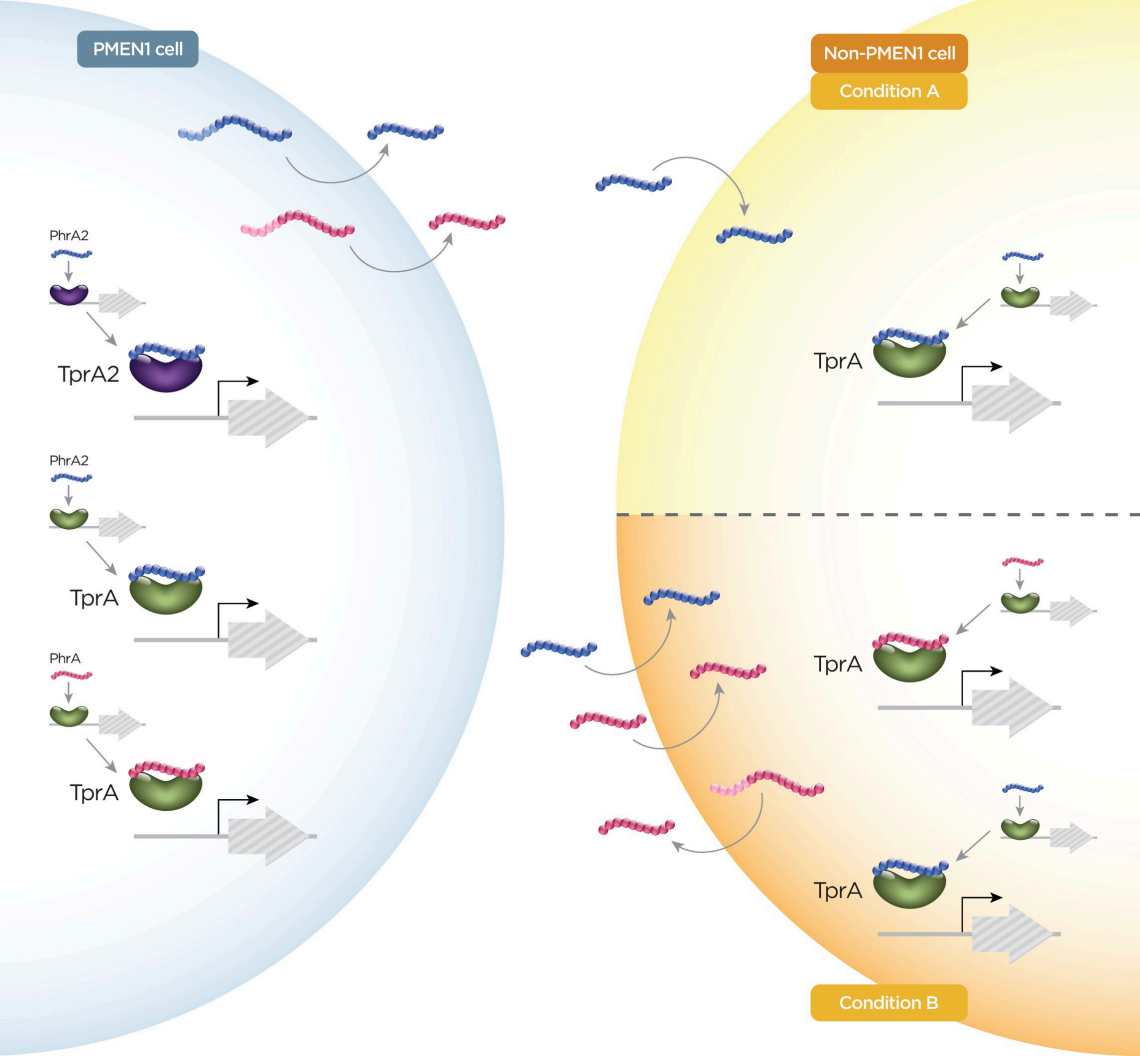
Example of peptide crosstalk, as seen for PhrA and PhrA2. The blue cell on the left side of the diagram encodes both TprA/PhrA and TprA2/PhrA2. The yellow cell on the right side of the diagram encodes only for TprA/PhrA. The gene encoding PhrA is induced in galactose and repressed in glucose; its promoter encodes a site for CcpA catabolite repression. In contrast, *phrA2* is not repressed by glucose. Thus, when PMEN1 is grown in glucose, PhrA2 is produced. Moreover, PhrA2 can bind TprA, partially overcoming repression of the TprA/PhrA system. In accordance, PhrA is also produced in PMEN1 cells grown in glucose. Because PhrA2 is secreted and imported by the ubiquitous oligopeptide permease system, AmiACDEF, PhrA2 can bind TprA in neighboring cells, independent of strain identity. In vitro, PhrA2 secreted by PMEN1 cells activates gene expression of *tprA* in both PMEN1 and non-PMEN1 cells. This figure illustrates cells in rich media, where signaling of non-PMEN1 cells is reduced. In the top panel (condition A), in the absence of input from the neighboring PMEN1 strain, TprA is bound to DNA, inhibiting gene expression. In the lower panel (condition B), in the presence of peptides from the neighboring PMEN1 strain, TprA/PhrA system is induced by exogenous peptides, ultimately inducing production of endogenous PhrA. PMEN1, pneumococcal molecular epidemiology network clone 1.

Analogous to TprA/PhrA, PhrA2 interacts with TprA2 resulting in the derepression of the TprA2 regulon and induced expression of a lanthionine-containing peptide (LcpA), immediately downstream [44]. There is evidence of unidirectional crosstalk between the TprA2/PhrA2 and the TprA/PhrA systems **(Fig 3)** [44]. Presence of PhrA2 activates signaling through the TprA/PhrA system resulting in an induction of the TprA regulon. In contrast, the presence of PhrA does not activate the expression of TprA2 regulon. Interestingly, PhrA2 from a PMEN1 strain can relieve the inhibition of the noncognate regulator TprA in a non-PMEN1 strain. PhrA is normally induced in the presence of galactose, while PhrA2 regulation has not been shown to be dependent on carbon source [26,46]. This crosstalk may allow the induction of PhrA in conditions where PhrA2 is induced and galactose is not the main carbon source. This interaction highlights the potential of the Tpr/Phr systems to influence interstrain communication in multistrain colonization events.

## Perspectives and conclusions

The diversity of peptides encoded by the pneumococcus highlights the importance of community-level phenotypes in introducing genetic diversity, microbial competition, and environmental adaptation. Several studies demonstrated the contribution of intercellular communication in pneumococcal virulence. Additional studies are required to map the spatiotemporal expression of these systems during the course of infection.

The upper respiratory tract is the resident niche for many bacterial species [104]. To establish itself in the host environment, pneumococcal cells must survive the competition and resist challenges mounted by other resident species. Pneumococci produce numerous peptides that mediate bactericidal activity either directly (e.g., Blp peptides or lantibiotics) or indirectly by activating downstream molecules (e.g., activation of CibAB or CbpD by CSP). While some of these bacteriocins are characterized, many putative bacteriocins await functional characterization, especially in the context of target strains/species and environmental conditions that stimulate their synthesis.

Multiple pneumococcal peptides are associated with biofilm development, including CSP, BriC, and VP1. Biofilms play a critical role in carriage and disease [77,105–110]. Carriage includes a biofilm mode of growth, which itself enables local cell–cell communications (by peptides and AI-2) and uptake of DNA supplied in the biofilm matrix. Further, biofilms provide a platform for phenotypic heterogeneity, an underexplored topic, perhaps contributing to pneumococcal fitness in chronic infections and adaptation to host niche. In addition, biofilms promote pathogenesis. They not only serve as a ground from which bacteria can disseminate but also bacteria disseminated from biofilm are more virulent than their biofilm or planktonic counterparts [111]. In summary, pneumococcal signaling peptides and biofilms are tightly connected: local environments in biofilms likely promote conditions for cell–cell communication and peptides to influence biofilm development and dispersion to other tissues and new hosts.

The control of regulatory networks by peptides allows the pneumococcus to quickly respond to its environment, not only at the level of an individual cell but also at the level of the entire population. Crosstalk between systems and regulation of a pathway by multiple peptides allows the different signals to be integrated, presumably increasing the dynamics and complexity of responses. Studies have revealed that multistrain pneumococcal co-colonization is a relatively common occurrence [112–115], highlighting the importance of peptide exchange across strains. Crosstalk has been documented for PhrA2 and PhrA (**Fig 3**). Further, peptides from the same family or diverse families can signal in the same pathway. For instance, signaling through CSP induces multiple double-glycine proteins, and SHP144 induces the levels of VP1, a double-glycine peptide. This hierarchy of peptide activation depicts the existence of multiple points of entry into activation of biological pathways. It indicates that the activation of these pathways is tightly controlled and represents related but different possibilities. One possibility is that different signal combinations allow for induction of an entire pathway or section(s) of the pathway, providing granularity in the type, magnitude, and metabolic costs associated with a response. Alternatively, the cell may activate peptide signaling to prepare itself for the induction of downstream pathways upon encountering suitable conditions.

Antibiotic resistance is a global public health concern; hence, there is an urgent need to develop effective anti-infectives. Nonantibiotic anti-infectives are expected to reduce the emergence of antibiotic resistance. Different strategies being used to develop compounds that will disrupt cell–cell communication include inhibition of signal production, signal degradation, or blocking signal transduction [116,117]. The use of chemical compounds has been shown to disrupt Rgg/SHP signaling and consequently biofilm development in *S*. *pyogenes* and some other streptococcal species [118]. In pneumococcus, the use of competitive analogs to CSP has been shown to inhibit competence development and horizontal gene transfer [119]. Further, anti-infectives in the form of soluble LMIP that target PhrA peptides decreased pneumococcus-mediated morbidity in mice [45]. How the different anti-infectives influence bacterial fitness and contribute to resistance development remains to be tested. The diversity of cell–cell communication systems in pneumococcus provides numerous opportunities to explore the different possibilities. The study of community level phenotype of *S*. *pneumoniae* provides opportunities for developing novel anti-infectives targeting peptide-mediated systems as well as the pathways modulated by these systems.

## Supporting information

S1 Table Protein sequences for the pneumococcal peptides described in this article. (XLSX)

## References

[1] Fuqua WC, Winans SC, Greenberg EP. Quorum sensing in bacteria: The LuxR-LuxI family of cell density- responsive transcriptional regulators. J Bacteriol. 1994;176:269–275. 10.1128/jb.176.2.269-275.1994 8288518 PMC205046

[2] Nealson KH. Autoinduction of Bacterial Luciferase. Arch Microbiol. 1977;112:73–79. 10.1007/BF00446657 843170

[3] Kaiser D, Losick R. How and why bacteria talk to each other. Cell. 1993;73:873–885. 10.1016/0092-8674(93)90268-u 8500179

[4] Kaiser D. Bacteria Also Vote Science. 1996;272:1598–1599. 10.1126/science.272.5268.1598 8658131

[5] Gray KM. Intercellular communication and group behaviour in bacteria. Trends Microbiol. 1997;5:184–188. 10.1016/S0966-842X(97)01002-0 9160506

[6] Bassler BL. Small talk: Cell-to-cell communication in bacteria. Cell. 2002;109:421–424. 10.1016/s0092-8674(02)00749-3 12086599

[7] Lancet. Acute respiratory infections in under-fives: 15 million deaths a year. Lancet. 1985;326:699–701. 2863681

[8] O’Brien KL, Wolfson LJ, Watt JP, Henkle E, Deloria-Knoll M, McCall N et al Burden of disease caused by *Streptococcus pneumoniae* in children younger than 5 years: global estimates. Lancet. 2009;374:893–902. 10.1016/S0140-6736(09)61204-6 19748398

[9] GBD 2016 Lower Respiratory Infections Collaborators. Estimates of the global, regional, and national morbidity, mortality, and aetiologies of lower respiratory infections in 195 countries, 1990–2016: a systematic analysis for the Global Burden of Disease Study 2016. Lancet Infect Dis. 2018;18:1191–1210. 10.1016/S1473-3099(18)30310-4 30243584 PMC6202443

[10] Sadé J, Luntz M, Levy D. Middle ear gas composition and middle ear aeration. Ann Otol Rhinol Laryngol. 1995;104:369–373. 10.1177/000348949510400506 7747907

[11] Harell M, Mover-Lev H, Levy D, Sadé J. Gas composition of the human nose and nasopharyngeal space. Acta Otolaryngol. 1996;116:82–84. 10.3109/00016489609137718 8820356

[12] Hergils L, Magnuson B. Middle ear gas composition in pathologic conditions: Mass spectrometry in otitis media with effusion and atelectasis. Ann Otol Rhinol Laryngol. 1997;106:743–745. 10.1177/000348949710600905 9302904

[13] McDevitt CA, Ogunniyi AD, Valkov E, Lawrence MC, Kobe B, McEwan AG et al A molecular mechanism for bacterial susceptibility to Zinc. PLoS Pathog. 2011;7:e1002357 10.1371/journal.ppat.1002357 22072971 PMC3207923

[14] King SJ, Hippe KR, Weiser JN. Deglycosylation of human glycoconjugates by the sequential activities of exoglycosidases expressed by *Streptococcus pneumoniae*. Mol Microbiol. 2006;59:961–974. 10.1111/j.1365-2958.2005.04984.x 16420364

[15] Yesilkaya H, Manco S, Kadioglu A, Terra VS, Andrew PW. The ability to utilize mucin affects the regulation of virulence gene expression in *Streptococcus pneumoniae*. FEMS Microbiol Lett. 2008;278:231–235. 10.1111/j.1574-6968.2007.01003.x 18053067

[16] Webb P. Air temperatures in respiratory tracts of resting subjects in cold. J Appl Physiol. 1951;4:378–382. 10.1152/jappl.1951.4.5.378 14938268

[17] Hine K, Hosono S, Kawabata K, Miyabayashi H, Kanno K, Shimizu M et al Nasopharynx is well-suited for core temperature measurement during hypothermia therapy. Pediatr Int. 2017;59:29–33. 10.1111/ped.13046 27273561

[18] Havarstein LS, Coomaraswamy G, Morrison DA. An unmodified heptadecapeptide pheromone induces competence for genetic transformation in *Streptococcus pneumoniae*. Proc Natl Acad Sci U S A. 1995;92:11140–11144. 10.1073/pnas.92.24.11140 7479953 PMC40587

[19] de Saizieu A, Gardes C, Flint N, Wagner C, Kamber M, Mitchell TJ et al Microarray-based identification of a novel *Streptococcus pneumoniae* regulon controlled by an autoinduced peptide. J Bacteriol. 2000;182:4696–4703. 10.1128/jb.182.17.4696-4703.2000 10940007 PMC111343

[20] Guiral S, Mitchell TJ, Martin B, Claverys J-PP. Competence-programmed predation of noncompetent cells in the human pathogen *Streptococcus pneumoniae*: Genetic requirements. Proc Natl Acad Sci U S A. 2005;102:8710–8715. 10.1073/pnas.0500879102 15928084 PMC1150823

[21] Son MR, Shchepetov M, Adrian PV, Madhi SA, de Gouveia L, von Gottberg A et al Conserved mutations in the pneumococcal bacteriocin transporter gene, *blpA*, result in a complex population consisting of producers and cheaters. MBio. 2011;2:e00179–e00111. 10.1128/mBio.00179-11 21896678 PMC3171984

[22] Cuevas RA, Eutsey R, Kadam A, West-Roberts JA, Woolford CA, Mitchell AP et al A novel streptococcal cell–cell communication peptide promotes pneumococcal virulence and biofilm formation. Mol Microbiol. 2017;105:554–571. 10.1111/mmi.13721 28557053 PMC5550342

[23] Aggarwal SD, Eutsey R, West-Roberts J, Domenech A, Xu W, Abdullah IT et al Function of BriC peptide in the pneumococcal competence and virulence portfolio. PLoS Pathog. 2018;14:e1007328 10.1371/journal.ppat.1007328 30308062 PMC6181422

[24] Aprianto R, Slager J, Holsappel S. Veening J. High-resolution analysis of the pneumococcal transcriptome under a wide range of infection-relevant conditions. 2018;46:9990–10006. 10.1093/nar/gky750 30165663 PMC6212715

[25] Wang CY, Medlin JS, Nguyen DR, Disbennett WM, Dawid S. Molecular determinants of substrate selectivity of a pneumococcal Rgg-regulated peptidase-containing ABC transporter. MBio. 2020;11:e02502–e02519. 10.1128/mBio.02502-19 32047125 PMC7018657

[26] Hoover SE, Perez AJ, Tsui HCT, Sinha D, Smiley DL, Dimarchi RD et al A new quorum-sensing system (TprA/PhrA) for *Streptococcus pneumoniae* D39 that regulates a lantibiotic biosynthesis gene cluster. Mol Microbiol. 2015;97:229–243. 10.1111/mmi.13029 25869931 PMC4676566

[27] Javan RR, van Tonder AJ, King JP, Harrold CL, Brueggemann AB. Genome sequencing reveals a large and diverse repertoire of antimicrobial peptides. Front Microbiol. 2018;9:2012 10.3389/fmicb.2018.02012 30210481 PMC6120550

[28] Havarstein LS, Holo H, Nes IF. The leader peptide of colicin V shares consensus sequences with leader peptides that are common among peptide bacteriocins produced by Gram-positive bacteria. Microbiology. 1994;140:2383–2389. 10.1099/13500872-140-9-2383 7952189

[29] Havarstein LS, Diep DB, Nes IF. A family of bacteriocin ABC transporters carry out proteolytic processing of their substrates concomitant with export. Mol Microbiol. 1995;16:229–240. 10.1111/j.1365-2958.1995.tb02295.x 7565085

[30] Hui FM, Zhou L, Morrison DA. Competence for genetic transformation in *Streptococcus pneumoniae*: organization of a regulatory locus with homology to two lactococcin A secretion genes. Gene. 1995;153:25–31. 10.1016/0378-1119(94)00841-f 7883181

[31] Dawid S, Roche AM, Weiser JN. The *blp* bacteriocins of *Streptococcus pneumoniae* mediate intraspecies competition both in vitro and in vivo. Infect Immun. 2007;75:443–451. 10.1128/IAI.01775-05 17074857 PMC1828380

[32] Kjos M, Miller E, Slager J, Lake FB, Gericke O, Roberts IS et al Expression of Streptococcus pneumoniae bacteriocins Is induced by antibiotics via regulatory interplay with the competence system. PLoS Pathog. 2016;12:e1005422 10.1371/journal.ppat.1005422 26840404 PMC4739728

[33] Wholey W-Y, Kochan TJ, Storck DN, Dawid S. Coordinated bacteriocin expression and competence in *Streptococcus pneumoniae* contributes to genetic adaptation through neighbor predation. PLoS Pathog. 2016;12:e1005413 10.1371/journal.ppat.1005413 26840124 PMC4739721

[34] Wang CY, Patel N, Wholey W-Y, Dawid S. ABC transporter content diversity in *Streptococcus pneumoniae* impacts competence regulation and bacteriocin production. Proc Natl Acad Sci. 2018;115:E5776–E5785. 10.1073/pnas.1804668115 29866828 PMC6016807

[35] Pestova EV, Håvarstein LS, Morrison DA. Regulation of competence for genetic transformation in *Streptococcus pneumoniae* by an auto-induced peptide pheromone and a two-component regulatory system. Mol Microbiol. 1996;21:853–862. 10.1046/j.1365-2958.1996.501417.x 8878046

[36] Wholey W-Y, Abu-Khdeir M, Yu EA, Siddiqui S, Esimai O, Dawid S. Characterization of the competitive pneumocin peptides of *Streptococcus pneumoniae*. Front Cell Infect Microbiol. 2019;9:55 10.3389/fcimb.2019.00055 30915281 PMC6422914

[37] Pundir P, Liu R, Vasavda C, Serhan N, Limjunyawong N, Yee R et al A Connective tissue mast-cell-specific receptor detects bacterial quorum-sensing molecules and mediates antibacterial immunity. Cell Host Microbe. 2019;26:114–122. 10.1016/j.chom.2019.06.003 31278040 PMC6649664

[38] Declerck N, Bouillaut L, Chaix D, Rugani N, Slamti L, Hoh F et al Structure of PlcR: Insights into virulence regulation and evolution of quorum sensing in Gram-positive bacteria. Proc Natl Acad Sci U S A. 2007;104:18490–18495. 10.1073/pnas.0704501104 17998541 PMC2141804

[39] Mashburn-Warren L, Morrison DA, Federle MJ. A novel double-tryptophan peptide pheromone controls competence in Streptococcus spp. via an Rgg regulator. Mol Microbiol. 2010;78:589–606. 10.1111/j.1365-2958.2010.07361.x 20969646 PMC3058796

[40] Fleuchot B, Gitton C, Guillot A, Vidic J, Nicolas P, Besset C et al Rgg proteins associated with internalized small hydrophobic peptides: A new quorum-sensing mechanism in streptococci. Mol Microbiol. 2011;80:1102–1119. 10.1111/j.1365-2958.2011.07633.x 21435032

[41] Cook LC, Federle MJ. Peptide pheromone signaling in Streptococcus and Enterococcus. FEMS Microbiol Rev. 2014;38:473–492. 10.1111/1574-6976.12046 24118108 PMC4103628

[42] Junges R, Salvadori G, Shekhar S, Åmdal HA, Periselneris JN. Chen T, et al A quorum-sensing system that regulates *Streptococcus pneumoniae* biofilm formation and surface polysaccharide production mSphere. 2017;2:e00324–e00317. 10.1128/mSphere.00324-17 28932816 PMC5597970

[43] Zhi X, Abdullah IT, Gazioglu O, Manzoor I, Shafeeq S, Kuipers OP et al Rgg-Shp regulators are important for pneumococcal colonization and invasion through their effect on mannose utilization and capsule synthesis. Sci Rep. 2018;8:6369 10.1038/s41598-018-24910-1 29686372 PMC5913232

[44] Kadam A, Eutsey RA, Rosch J, Miao X, Longwell M, Xu W, et al Promiscuous signaling by a regulatory system unique to the pandemic PMEN1 pneumococcal lineage. Orihuela CJ, editor. PLoS Pathog 2017;13: e1006339 10.1371/journal.ppat.1006339 28542565 PMC5436883

[45] Motib A, Guerreiro A, Al-Bayati F, Piletska E, Manzoor I, Shafeeq S et al Modulation of quorum sensing in a Gram-positive pathogen by linear molecularly imprinted polymers with anti-infective properties. Angew Chemie—Int Ed. 2017;56:16555–16558. 10.1002/anie.201709313 29140595

[46] Motib AS, Al-Bayati FAY, Manzoor I, Shafeeq S, Kadam A, Kuipers OP et al TprA/PhrA quorum sensing system has a major effect on pneumococcal survival in respiratory tract and blood, and its activity is controlled by CcpA and GlnR. Front Cell Infect Microbiol. 2019;9:326 10.3389/fcimb.2019.00326 31572692 PMC6753895

[47] Ibrahim M, Guillot A, Wessner F, Algaron F, Besset C, Courtin P et al Control of the transcription of a short gene encoding a cyclic peptide in *Streptococcus thermophilus*: A new quorum-sensing system? J Bacteriol. 2007;189:8845–8854. 10.1128/JB.01057-07 17921293 PMC2168622

[48] Fleuchot B, Guillot A, Mézange C, Besset C, Chambellon E, Monnet V et al Rgg-associated SHP signaling peptides mediate cross-talk in Streptococci. PLoS One. 2013;8:e66042 10.1371/journal.pone.0066042 23776602 PMC3679016

[49] Aggarwal C, Jimenez JC, Nanavati D, Federle MJ. Multiple length peptide-pheromone variants produced by *Streptococcus pyogenes* directly bind Rgg proteins to confer transcriptional regulation. J Biol Chem. 2014;289:22427–22436. 10.1074/jbc.M114.583989 24958729 PMC4139249

[50] Chang JC, LaSarre B, Jimenez JC, Aggarwal C, Federle MJ. Two group A streptococcal peptide pheromones act through opposing Rgg regulators to control biofilm development. PLoS Pathog. 2011;7:e1002190 10.1371/journal.ppat.1002190 21829369 PMC3150281

[51] Varahan S, Harms N, Gilmore MS, Tomich JM, Hancock LE. An ABC transporter is required for secretion of peptide sex pheromones in *Enterococcus faecalis*. MBio. 2014;5:e01726–e01714. 10.1128/mBio.01726-14 25249282 PMC4173765

[52] Pérez-Pascual D, Gaudu P, Fleuchot B, Besset C, Rosinski-Chupin I, Guillot A et al RovS and its associated signaling peptide form a cell-to-cell communication system required for *Streptococcus agalactiae* pathogenesis. MBio. 2015;6:e02306–e02314. 10.1128/mBio.02306-14 25604789 PMC4324310

[53] Chang JC, Federle MJ. PptAB exports Rgg quorum-sensing peptides in Streptococcus. PLoS One. 2016;11:e0168461 10.1371/journal.pone.0168461 27992504 PMC5167397

[54] Perego M, Hoch JA. Cell-cell communication regulates the effects of protein aspartate phosphatases on the phosphorelay controlling development in *Bacillus subtilis*. Proc Natl Acad Sci U S A. 1996;93:1549–1553. 10.1073/pnas.93.4.1549 8643670 PMC39978

[55] Rocha-Estrada J, Aceves-Diez AE, Guarneros G, De La Torre M. The RNPP family of quorum-sensing proteins in Gram-positive bacteria. Appl Microbiol Biotechnol. 2010;87:913–923. 10.1007/s00253-010-2651-y 20502894

[56] Pottathil M, Lazazzera BA. The extracellular Phr peptide-Rap phosphatase signaling circuit of *Bacillus subtilis*. Front Biosci. 2003;8:d32–d45. 10.2741/913 12456319

[57] Solomon JM, Lazazzera BA, Grossman AD. Purification and characterization of an extracellular peptide factor that affects two different developmental pathways in *Bacillus subtilis*. Genes Dev. 1996;10:2014–2024. 10.1101/gad.10.16.2014 8769645

[58] Perego M. A peptide export-import control circuit modulating bacterial development regulates protein phosphatases of the phosphorelay. Proc Natl Acad Sci U S A. 1997;94:8612–8617. 10.1073/pnas.94.16.8612 9238025 PMC23044

[59] Lazazzera BA, Solomon JM. Grossman AD. An exported peptide functions intracellularly to contribute to cell density signaling in *B subtilis* Cell. 1997;89:917–925. 10.1016/S0092-8674(00)80277-9 9200610

[60] Song XM, Connor W, Hokamp K, Babiuk LA, Potter AA. Transcriptome studies on *Streptococcus pneumoniae*, illustration of early response genes to THP-1 human macrophages. Genomics. 2009;93:72–82. 10.1016/j.ygeno.2008.09.008 18848982

[61] Mirouze N, Parashar V, Baker MD, Dubnau DA, Neiditch MB. An atypical Phr peptide regulates the developmental switch protein RapH. J Bacteriol. 2011;193:6197–6206. 10.1128/JB.05860-11 21908671 PMC3209236

[62] Willey JM, van der Donk WA. Lantibiotics: Peptides of diverse structure and function. Annu Rev Microbiol. 2007;61:477–501. 10.1146/annurev.micro.61.080706.093501 17506681

[63] Sen AK, Narbad A, Horn N, Dodd HM, Parr AJ, Colquhoun I et al Post-translational modification of nisin. Eur J Biochem. 1999;261:524–532. 10.1046/j.1432-1327.1999.00303.x 10215865

[64] Koponen O, Tolonen M, Qiao M, Wahlström G, Helin J, Saris PEJ. NisB is required for the dehydration and NisC for the lanthionine formation in the post-translational modification of nisin. Microbiology. 2002;148:3561–3568. 10.1099/00221287-148-11-3561 12427947

[65] Begley M, Cotter PD, Hill C, Ross RP. Identification of a novel two-peptide lantibiotic, lichenicidin, following rational genome mining for LanM proteins. Appl Environ Microbiol. 2009;75:5451–5460. 10.1128/AEM.00730-09 19561184 PMC2737927

[66] Maricic N, Anderson ES, Opipari AE, Yu EA, Dawid S. Characterization of a multipeptide lantibiotic locus in *Streptococcus pneumoniae*. MBio. 2016;7:e01656–e01615. 10.1128/mBio.01656-15 26814178 PMC4742701

[67] Tomasz A. Control of the competent state in pneumococcus by a hormone-like cell product: An example for a new type of regulatory mechanism in bacteria. Nature. 1965;208:155–159. 10.1038/208155a0 5884251

[68] Chen J-D, Morrison DA. Modulation of competence for genetic transformation in *Streptococcus pneumoniae*. J Gen Microbiol. 1987;133:1959–1967. 10.1099/00221287-133-7-1959 3668504

[69] Echenique JR, Chapuy-Regaud S, Trombe MC. Competence regulation by oxygen in *Streptococcus pneumoniae*: involvement of *ciaRH* and *comCDE*. Mol Microbiol. 2000;36:688–696. 10.1046/j.1365-2958.2000.01891.x 10844657

[70] Claverys J-P, Havarstein LS. Extracellular-peptide control of competence for genetic transformation in *Streptococcus pneumoniae*. Front Biosci. 2002;7:1798–1814. 10.2741/claverys 12133809

[71] Prudhomme M, Attaiech L, Sanchez G, Martin B, Claverys J-P. Antibiotic stress induces genetic transformability in the human pathogen *Streptococcus pneumoniae*. Science. 2006;313:89–92. 10.1126/science.1127912 16825569

[72] Gagne AL, Stevens KE, Cassone M, Pujari A, Abiola OE, Chang DJ et al Competence in *Streptococcus pneumoniae* is a response to an increasing mutational burden. PLoS One. 2013;8:e72613 10.1371/journal.pone.0072613 23967325 PMC3742669

[73] Slager J, Kjos M, Attaiech L, Veening JW. Antibiotic-induced replication stress triggers bacterial competence by increasing gene dosage near the origin. Cell. 2014;157:395–406. 10.1016/j.cell.2014.01.068 24725406

[74] Domenech A, Slager J, Veening JW. Antibiotic-induced cell chaining triggers pneumococcal competence by reshaping quorum sensing to autocrine-like signaling. Cell Rep. 2018;25:2390–2400. 10.1016/j.celrep.2018.11.007 30485808 PMC6289044

[75] Peterson SN, Sung CK, Cline R, Desai BV, Snesrud EC, Luo P et al Identification of competence pheromone responsive genes in *Streptococcus pneumoniae* by use of DNA microarrays. Mol Microbiol. 2004;51:1051–1070. 10.1046/j.1365-2958.2003.03907.x 14763980

[76] Claverys J-P, Prudhomme M, Martin B. Induction of competence regulons as a general response to stress in Gram-positive bacteria. Annu Rev Microbiol. 2006;60:451–475. 10.1146/annurev.micro.60.080805.142139 16771651

[77] Oggioni MR, Trappetti C, Kadioglu A, Cassone M, Iannelli F, Ricci S et al Switch from planktonic to sessile life: A major event in pneumococcal pathogenesis. Mol Microbiol. 2006;61:1196–1210. 10.1111/j.1365-2958.2006.05310.x 16925554 PMC1618759

[78] Trappetti C, Gualdi L. Meola L Di, Jain P, Korir CC, Edmonds P, et al The impact of the competence quorum sensing system on Streptococcus pneumoniae biofilms varies depending on the experimental model BMC Microbiol. 2011;11:75 10.1186/1471-2180-11-75 21492426 PMC3098770

[79] Cuevas RA, Ebrahimi E, Gazioglu O, Yesilkaya H, Hiller NL. Pneumococcal attachment to epithelial cells is enhanced by the secreted peptide VP1 via its control of hyaluronic acid processing. bioRxiv. 2019 10.1101/788430

[80] Howard LM, Zhu Y, Griffin MR, Edwards KM, Williams JV, Gil AI et al Nasopharyngeal pneumococcal density during asymptomatic respiratory virus infection and risk for subsequent acute respiratory illness. Emerg Infect Dis. 2019;25:2040–2047. 10.3201/eid2511.190157 31625844 PMC6810199

[81] Whatmore AM, Barcus VA, Dowson CG. Genetic diversity of the streptococcal competence (*com*) gene locus. J Bacteriol. 1999;181:3144–3154. 10.1128/JB.181.10.3144-3154.1999 10322016 PMC93770

[82] Yang J, Evans BA, Rozen DE. Signal diffusion and the mitigation of social exploitation in pneumococcal competence signalling. Proc R Soc B Biol Sci. 2010;277:2991–2999. 10.1098/rspb.2010.0659 20462905 PMC2982029

[83] Prudhomme M, Berge M, Martin B, Polard P. Pneumococcal competence coordination relies on a cell-contact sensing mechanism. PLoS Genet. 2016;12:1–24. 10.1371/journal.pgen.1006113 27355362 PMC4927155

[84] Dagkessamanskaia A, Moscoso M, Overweg K, Reuter M, Martin B, Wells J et al Interconnection of competence, stress and CiaR regulons in *Streptococcus pneumoniae*: competence triggers stationary phase autolysis of *ciaR* mutant cells. Mol Microbiol. 2004;51:1071–1086. 10.1111/j.1365-2958.2003.03892.x 14763981

[85] Cooper VS, Honsa E, Rowe H, Deitrick C, Iverson AR. Whittall JJ, et al Experimental evolution *in vivo* to identify selective pressures during pneumococcal colonization mSystems. 2020;5:e00352–e00320. 10.1128/mSystems.00352-20 32398278 PMC7219553

[86] Martinez-Cuesta MC, Kok J, Herranz E, Pelaez C, Requena T, Buist G. Requirement of autolytic activity for bacteriocin-induced lysis. Appl Environ Microbiol. 2000;66:3174–3179. 10.1128/aem.66.8.3174-3179.2000 10919766 PMC92130

[87] Claverys JP, Håvarstein LS. Cannibalism and fratricide: Mechanisms and raisons d’être. Nat Rev Microbiol. 2007;5:219–229. 10.1038/nrmicro1613 17277796

[88] Shen P, Lees JA, Bee GCW, Brown SP, Weiser JN. Pneumococcal quorum sensing drives an asymmetric owner-intruder competitive strategy during carriage via the competence regulon. Nat Micrbiology. 2019;4:198–208. 10.1016/j.physbeh.2017.03.040 30546100 PMC6342471

[89] Reichmann P, Hakenbeck R. Allelic variation in a peptide-inducible two-component system of *Streptococcus pneumoniae*. FEMS Microbiol Lett. 2000;190:231–236. 10.1111/j.1574-6968.2000.tb09291.x 11034284

[90] Bogaardt C, van Tonder AJ, Brueggemann AB. Genomic analyses of pneumococci reveal a wide diversity of bacteriocins—including pneumocyclicin, a novel circular bacteriocin. BMC Genomics. 2015;16:554 10.1186/s12864-015-1729-4 26215050 PMC4517551

[91] Miller EL, Abrudan MI, Roberts IS, Rozen DE. Diverse ecological strategies are encoded by *Streptococcus pneumoniae* bacteriocin-like peptides. Genome Biol Evol. 2016;8:1072–1090. 10.1093/gbe/evw055 26983823 PMC4860687

[92] Pinchas MD, LaCross NC, Dawid S. An electrostatic interaction between BlpC and BlpH dictates pheromone specificity in the control of bacteriocin production and immunity in *Streptococcus pneumoniae*. J Bacteriol. 2015;197:1236–1248. 10.1128/JB.02432-14 25622617 PMC4352655

[93] Paixão L, Oliveira J, Veríssimo A, Vinga S, Lourenço EC, Ventura MR et al Host glycan sugar-specific pathways in *Streptococcus pneumonia*: Galactose as a key sugar in colonisation and infection. PLoS One. 2015;10:e0121042 10.1371/journal.pone.0121042 25826206 PMC4380338

[94] Higgins MA, Suits MD, Marsters C, Boraston AB. Structural and functional analysis of fucose-processing enzymes from *Streptococcus pneumoniae*. J Mol Biol. 2014;426:1469–1482. 10.1016/j.jmb.2013.12.006 24333485

[95] Deutscher J, Francke C, Postma PW. How phosphotransferase system-related protein phosphorylation regulates carbohydrate metabolism in bacteria. Microbiol Mol Biol Rev. 2006;70:939–1031. 10.1128/MMBR.00024-06 17158705 PMC1698508

[96] Sundar GS, Islam E, Gera K, Le Breton Y, McIver KS. A PTS EII mutant library in Group A Streptococcus identifies a promiscuous man-family PTS transporter influencing SLS-mediated hemolysis. Mol Microbiol. 2017;103:518–533. 10.1111/mmi.13573 27862457 PMC5263093

[97] Hendriksen WT, Bootsma HJ, Estevão S, Hoogenboezem T, De Jong A, De Groot R et al CodY of *Streptococcus pneumoniae*: Link between nutritional gene regulation and colonization. J Bacteriol. 2008;190:590–601. 10.1128/JB.00917-07 18024519 PMC2223708

[98] Hendriksen WT, Kloosterman TG, Bootsma HJ, Estevão S, de Groot R, Kuipers OP et al Site-specific contributions of glutamine-dependent regulator GlnR and GlnR-regulated genes to virulence of *Streptococcus pneumoniae*. Infect Immun. 2008;76:1230–1238. 10.1128/IAI.01004-07 18174343 PMC2258823

[99] Junges R, Sturød K, Salvadori G, Åmdal HA, Chen T, Petersen FC. Characterization of a signaling system in *Streptococcus mitis* that mediates interspecies communication with *Streptococcus pneumoniae*. Appl Environ Microbiol Microbiol. 2019;85:e02297–e02218. 10.1128/AEM.02297-18 30389765 PMC6328782

[100] Marion C, Stewart JM, Tazi MF, Burnaugh AM, Linke CM, Woodiga SA et al *Streptococcus pneumoniae* can utilize multiple sources of hyaluronic acid for growth. Infect Immun. 2012;80:1390–1398. 10.1128/IAI.05756-11 22311922 PMC3318431

[101] Yadav MK, Chae SW, Park K, Song JJ. Hyaluronic acid derived from other streptococci supports *Streptococcus pneumoniae in vitro* biofilm formation. Biomed Res Int. 2013;2013:690217 10.1155/2013/690217 24171169 PMC3792519

[102] Carvalho SM, Kloosterman TG, Kuipers OP, Neves AR. CcpA ensures optimal metabolic fitness of *Streptococcus pneumoniae*. PLoS One. 2011;6:e26707 10.1371/journal.pone.0026707 22039538 PMC3198803

[103] van Opijnen T, Camilli A. A fine scale phenotype-genotype virulence map of a bacterial pathogen. Genome Res. 2012;22:2541–2551. 10.1101/gr.137430.112 22826510 PMC3514683

[104] Bosch AATM De Steenhuijsen Piters WAA, Van Houten MA Chu MLJN, Biesbroek G, Kool J et al Maturation of the infant respiratory microbiota, environmental drivers, and health consequences. Am J Respir Crit Care Med. 2017;196:1582–1590. 10.1164/rccm.201703-0554OC 28665684

[105] Hall-Stoodley L, Hu FZ, Gieseke A, Nistico L, Nguyen D, Hayes J et al Direct detection of bacterial biofilms on the middle-ear mucosa of children with chronic otitis media. JAMA. 2006;296:202–211. 10.1001/jama.296.2.202 16835426 PMC1885379

[106] Reid SD, Hong W, Dew KE, Winn DR, Pang B, Watt J et al *Streptococcus pneumoniae* forms surface-attached communities in the middle ear of experimentally infected chinchillas. J Infect Dis. 2009;199:786–794. 10.1086/597042 19434911

[107] Hoa M, Syamal M, Sachdeva L, Berk R, Coticchia J. Demonstration of nasopharyngeal and middle ear mucosal biofilms in an animal model of acute otitis media. Ann Otol Rhinol Laryngol. 2009;118:292–298. 10.1177/000348940911800410 19462851

[108] Sanderson AR, Leid JG, Hunsaker D. Bacterial biofilms on the sinus mucosa of human subjects with chronic rhinosinusitis. Laryngoscope. 2006;116:1121–1126. 10.1097/01.mlg.0000221954.05467.54 16826045

[109] Blanchette-Cain K, Hinojosa CA, Akula Suresh Babu R, Lizcano A, Gonzalez-Juarbe N, Munoz-Almagro C et al *Streptococcus pneumoniae* biofilm formation is strain dependent, multifactorial, and associated with reduced invasiveness and immunoreactivity during colonization. MBio. 2013;4:e00745–e00713. 10.1128/mBio.00745-13 24129258 PMC3812715

[110] Marks LR, Parameswaran GI, Hakansson AP. Pneumococcal interactions with epithelial cells are crucial for optimal biofilm formation and colonization in vitro and in vivo. Infect Immun. 2012;80:2744–2760. 10.1128/IAI.00488-12 22645283 PMC3434590

[111] Marks LR, Davidson BA, Knight PR, Hakansson AP. Interkingdom signaling induces *Streptococcus pneumoniae* biofilm dispersion and transition from asymptomatic colonization to disease. MBio. 2013;4:e00438–e00413. 10.1128/mBio.00438-13 23882016 PMC3735180

[112] Everett DB, Cornick J, Denis B, Chewapreecha C, Croucher N, Harris S et al Genetic characterisation of Malawian pneumococci prior to the roll-out of the PCV13 vaccine using a high-throughput whole genome sequencing approach. PLoS One. 2012;7:e44250 10.1371/journal.pone.0044250 22970189 PMC3438182

[113] Rodrigues F, Morales-Aza B, Turner KMEE, Sikora P, Gould K, Hinds J et al Multiple *Streptococcus pneumoniae* serotypes in aural discharge samples from children with acute otitis media with spontaneous otorrhea. J Clin Microbiol. 2013;51:3409–3411. 10.1128/JCM.01303-13 23885003 PMC3811614

[114] Saha SKS, Modak JK, Naziat H, Al-Emran HM, Chowdury M, Islam M et al Detection of co-colonization with *Streptococcus pneumoniae* by algorithmic use of conventional and molecular methods. Vaccine. 2015;33:713–718. 10.1016/j.vaccine.2014.11.040 25523524

[115] Chaguza C, Senghore M, Bojang E, Gladstone RA, Lo SW, Tientcheu PE et al Within-host microevolution of Streptococcus pneumoniae is rapid and adaptive during natural colonisation. Nat Commun. 2020;11:3442 10.1038/s41467-020-17327-w 32651390 PMC7351774

[116] Sintim HO, Smith JA, Wang J, Nakayama S, Yan L. Paradigm shift in discovering next-generation anti-infective agents: Targeting quorum sensing, c-di-GMP signaling and biofilm formation in bacteria with small molecules. Future Med Chem. 2010;2:1005–1035. 10.4155/fmc.10.185 21426116

[117] Hirakawa H, Tomita H. Interference of bacterial cell-to-cell communication: A new concept of antimicrobial chemotherapy breaks antibiotic resistance. Front Microbiol. 2013;4:114 10.3389/fmicb.2013.00114 23720655 PMC3652290

[118] Aggarwal C, Jimenez JC, Lee H, Chlipala GE, Ratia K, Federle MJ. Identification of quorum-sensing inhibitors disrupting signaling between Rgg and short hydrophobic peptides in streptococci. MBio. 2015;6:e00393–e00315. 10.1128/mBio.00393-15 25968646 PMC4436074

[119] Zhu L, Lau GW. Inhibition of competence development, horizontal gene transfer and virulence in *Streptococcus pneumoniae* by a modified competence stimulating peptide. PLoS Pathog. 2011;7:e1002241 10.1371/journal.ppat.1002241 21909280 PMC3164649

[120] Karlson P, Lüscher M. ‘Pheromones’: a new term for a class of biologically active substances. Nature. 1959;183:55–56. 10.1038/183055a0 13622694

[121] Iannelli F, Oggioni MR, Pozzi G. Sensor domain of histidine kinase ComD confers competence pherotype specificity in *Streptoccoccus pneumoniae*. FEMS Microbiol Lett. 2005;252:321–326. 10.1016/j.femsle.2005.09.008 16209911

[122] Håvarstein LS, Hakenbeck R, Gaustad P. Natural competence in the genus Streptococcus: Evidence that streptococci can change pherotype by interspecies recombinational exchanges. J Bacteriol. 1997;179:6589–6594. 10.1128/jb.179.21.6589-6594.1997 9352904 PMC179583

[123] Tortosa P, Dubnau D. Competence for transformation: A matter of taste. Curr Opin Microbiol. 1999;2:588–592. 10.1016/s1369-5274(99)00026-0 10607621

[124] Cornejo OE, McGee L, Rozen DE. Polymorphic competence peptides do not restrict recombination in *Streptococcus pneumoniae*. Mol Biol Evol. 2010;27:694–702. 10.1093/molbev/msp287 19942613

[125] Miller EL, Evans BA, Cornejo OE, Roberts IS, Rozen DE. Pherotype polymorphism in *Streptococcus pneumoniae* has no obvious effects on population structure and recombination. Genome Biol Evol. 2017;9:2546–2559. 10.1093/gbe/evx188 28992304 PMC5629823

